# The actual experiences of nurses’ in implementing hypothermia prevention practices during post-anesthesia care unit: a qualitative study based on the PRECEDE-PROCEED model

**DOI:** 10.3389/fmed.2025.1687814

**Published:** 2025-12-10

**Authors:** Jie Zou, Gongyin Luo, Yi Li

**Affiliations:** 1 Department of Nursing, Affiliated Hospital of Zunyi Medical University, Zunyi, Guizhou, China; 2 Department of Anesthesiology, Affiliated Hospital of Zunyi Medical University, Zunyi, Guizhou, China

**Keywords:** post-anesthesia care unit, hypothermia prevention practices, nurse, actual experiences qualitative research, PRECEDE-PROCEED model

## Abstract

**Background:**

Hypothermia is one of the most common postoperative complications in the post-anesthesia care unit (PACU), and preventive measures are crucial for improving patient outcomes. Anesthesia nurses play a vital role in the prevention and management of hypothermia, but the factors influencing their behavior have not yet been systematically explored.

**Objective:**

This study aimed to explore the actual experiences of the implementation in hypothermia prevention measures by PACU nurses in post-anesthesia care unit, providing a basis for the formulation of management plans.

**Methods:**

A descriptive qualitative research design was employed, with nurses in the post-anesthesia care unit of a general hospital in Guizhou Province, China, selected as the study subjects. Data analysis was performed directed content analysis.

**Results:**

Three major categories emerged, corresponding to the predisposing, reinforcing, and enabling factors outlined by the PRECEDE-PROCEED model. Predisposing factors included knowledge gaps on hypothermia, complexity of evidence-to-practice translation, conflicts between mindset and habitual practices. Enabling factors included insufficient human and equipment resources, lack of targeted and continuous training, lack of standardized guidelines for hypothermia prevention management processes, limitations in hospital information systems and lack of intelligent monitoring functions. Reinforcing factors included healthcare collaboration and peer support promote hypothermia prevention practices, lack of supervisory network for hypothermia prevention.

**Conclusion:**

To enhance hypothermia prevention practices in PACU, nursing administrators should implement need-based multidisciplinary training programs, optimize staffing, equipment resources, develop intelligent decision-support systems, and establish digitalized monitoring networks to support continuous quality improvement.

## 1 Introduction

The post-anesthesia care unit (PACU) plays a crucial role in monitoring and caring for patients following surgery until their vital signs stabilize, preventing postoperative complications during this period is essential for ensuring patient safety (1). Postoperative hypothermia (PH) is one of the most common complications observed in the PACU. Due to the effects of anesthesia and environmental factors, patients’ thermoregulation may be impaired, leading to PH (2). PH is defined as a core body temperature below 36.0°C upon admission to the next level of care (transition period between operating room and ward) after surgery (3). It is associated with numerous adverse outcomes, including coagulopathy, metabolic disorders, cardiovascular events, organ dysfunction, immunosuppression, increased risk of infection, and delayed emergence from anesthesia (4, 5). Additionally, shivering caused by PH can markedly increase oxygen consumption, thereby heightening the risk of myocardial ischemia, respiratory distress, and wound infection, ultimately delaying recovery, prolonging hospitalization, and increasing healthcare costs.

Studies report that the incidence of PH during PACU can be as high as 90% (6). Despite the issuance of various guidelines and consensus statements both domestically and internationally, the effectiveness of current hypothermia prevention efforts in the PACU remains suboptimal (7). As the implementers of intraoperative hypothermia prevention measures, nurses’ actions directly impact patient safety, and the quality of care during the anesthesia recovery period (8). Nurses are the primary implementers of safe and efficient perioperative care for patients, preventing adverse reactions (9). Existing research indicates that the prevention and management of peri-anesthetic hypothermia is not solely dependent on nurses’ individual behaviors, but is also influenced by multiple contributing factors. These include structural and environmental conditions; organizational and managerial elements such as staffing allocation and institutional support; and educational factors, namely the level of knowledge and competency among healthcare professionals regarding hypothermia prevention and management (10, 11). However, whether these factors affect anesthesia recovery room nurses’ implementation of hypothermia-related behaviors remains unclear. Therefore, comprehensively identifying and analyzing these multi-level determinants is essential for improving the quality of nursing care during the anesthesia recovery period. Due to methodological constraints, quantitative research tends to be limited in capturing and explaining the dynamic, contextualized subjective experiences and intrinsic motivational mechanisms underlying such behaviors (12). In contrast, qualitative research is more suitable for exploring the essence and meaning of specific phenomena as perceived by those directly involved.

Therefore, this study aimed to explore the actual experiences associated with the implementation of hypothermia-prevention behaviors among anesthesia nurses. It seeks to identify their authentic perceptions and lived experiences related to performing such preventive practices. The findings are expected to provide practical insights to strengthen nursing practice and ultimately reduce the incidence of hypothermia during PACU.

## 2 Materials and methods

### 2.1 Study design

This study employed a descriptive qualitative design (13),using the PRECEDE–PROCEED model as the guiding framework. Data were collected through in-depth interviews to capture participants’ internal experiences and perspectives, identify potential intervention leverage points, and help optimize strategies for health behavior management while improving behavioral outcomes. The PRECEDE–PROCEED model has been validated by multiple studies (14) as a useful theoretical framework for planning, implementing, and evaluating health promotion programs, and has been widely applied to explain how individual, psychological, and social determinants influence health-related behavioral change. Developed by Green and Kreuter (15), the model emphasizes not only the acquisition of health knowledge but also the developmental process of behavior change, categorizing determinants influencing the target behavior into predisposing, enabling, and reinforcing factors. Considering the potential influence of personal cultural background and contextual characteristics on the phenomenon under study, a directed content analysis approach (16) was applied to interpret and categorize the textual data. Consolidated Criteria for Reporting Qualitative Research (COREQ) were used to enhance its quality and transparency (17).

### 2.2 Participants and recruitment

This study employed purposive sampling to recruit nurses from PACU of a general hospital (This hospital is a provincial-level, grade A, and tertiary general hospital, its anesthesiology nursing staff can meet the requirements of this study) in Guizhou Province, China, between June and August 2025. Their profession, position, experience, and knowledge were also considered. Inclusion criteria were: (1) possessing a valid nursing license; (2) having at least one year of experience in PACU nursing; and (3) being capable of independently managing postoperative patient care. Exclusion criteria included: (1) being on leave (sick, personal, or maternity) for ≥ 3 months; (2) not having worked in PACU within the past year; and (3) being rotating or visiting nurses. The head nurse assisted in identifying research subjects who met the inclusion criteria. The study purpose was introduced in a meeting room setting, and nurses who initially expressed interest were invited to participate in a formal conversation outside of working hours. During these meetings, the researcher thoroughly explained the study objectives, procedures, potential benefits and risks, and emphasized that participation was entirely voluntary. It was clearly stated that refusal to participate or withdrawal at any stage would have no impact on their employment status or professional evaluation. All participants were given sufficient time to consider participation and ask questions. Written informed consent was obtained prior to enrollment and before conducting the first interview. The sample size was determined based on data saturation, defined as the point at which no new themes emerged, followed by an additional 2–3 interviews to confirm saturation (18). The study was conducted with full informed consent from participants and adhered to ethical principles regarding confidentiality and privacy protection.

### 2.3 Data collection and process

Data were collected through in-person, semi-structured interviews conducted in a quiet setting such as the PACU meeting room. Demographic data were completed by researchers using the review system. The interview guide was developed based on the PRECEDE-PROCEED framework, we developed a preliminary interview outline (JZ, GYL, and YL) through literature review (JZ and GYL) of relevant studies, group discussions, and expert consultation. Following pre-interviews with three anesthesia nurses, the outline was revised based on the interview process and outcomes to form the final interview guide. The final guide included questions such as: (1) What are the risks of postoperative hypothermia that you are aware of? (2) Do you think hypothermia management is necessary in the PACU? Why or why not? (3) How do you typically implement hypothermia prevention in your daily work? What factors hinder or facilitate your practice? (4) Which individuals and groups around you encourage or discourage you from implementing hypothermia prevention measures? (5) Have you received any training in hypothermia prevention? Was it effective? (6) Are you willing to change your previous operating habits? (7) Are you familiar with any guidelines or expert consensus statements on hypothermia management? Would you develop management strategies based on the temperature management recommendations in the guidelines or expert consensus statements? The interviews were conducted by two researchers following an interview guide. The lead researcher (an anesthesiology nurse with qualitative research experience and training in qualitative research and interview techniques) led the questioning and controlled the process, while the other researcher (an anesthesiology nurse manager trained in qualitative research and interview techniques) recorded non-verbal cues and posed follow-up questions during the latter part of the interview. During the interview, the researchers listened attentively to the participants’ narratives, avoiding leading language, commands, blame, or demanding tones. The interview concluded when the participant indicated no new information would emerge. Each interview lasted between 20 and 40 min.

### 2.4 Data analysis

To ensure data authenticity, the audio recordings were transcribed into text within 24 h without modification or deletion. The transcribed texts were imported into NVivo 11.0 software for systematic data management and analysis. Themes were identified through a six-stage thematic analysis (data familiarization, initial codes generation, searching for themes, carefully reviewing themes, defining and naming themes, and generating the report) (18). Two researchers (JZ and GYL) began by repeatedly reviewing the materials to grasp the overall context, and then independently performed line-by-line coding to identify initial concepts adopting inductive and deductive theme approaches. In order to produce the initial codes, each code was highlighted with a different color to identify the most significant and meaningful elements. Then, organize the code into meaningful categories, gradually inducting candidate classifications, sub-themes, and core themes. Deductive analysis was then conducted via PRECEDE-PROCEED framework to categorize the themes into the most relevant domains, with the results validated through multiple interviews. Any disagreements between the researchers during inductive coding and deductive categorization were resolved through discussion with a third researcher (YL) until consensus was achieved. An iterative approach was employed throughout the coding process, with the research team continually reviewing, revising, and refining the codes and thematic structure to enhance rigor and quality. Themes were developed from initial codes, whereas subthemes emerged from groupings of data with shared meanings. All the themes and subthemes were cross-checked against the original codes and transcripts. To ensure credibility and reliability, participant validation was carried out to confirm the findings.

### 2.5 Rigor and reflexivity

To ensure methodological rigor, this study adhered to the criteria of credibility, transferability, dependability, and confirmability as outlined by Lincoln and Guba (19) for trustworthiness and authenticity in naturalistic inquiry. Credibility was strengthened by employing semi-structured interviews and field observations, conducted by a postgraduate nursing researcher and a clinically experienced anesthesia nurse trained in qualitative research. Participants with varying lengths of professional experience were included to ensure sufficient depth and variation of the data. Transferability was supported through rich contextual descriptions, including participants’ sociodemographic characteristics, years of practice, experience in anesthesia recovery nursing, and the clinical context of PACU hypothermia (e.g., common triggers, workflow, and existing prevention practices). Additionally, the data collection procedures, interview guide domains, data organization and coding steps, and the roles of the research team were clearly documented to facilitate assessment of applicability to other institutions or settings. Dependability was ensured by developing a comprehensive audit trail, including interview transcripts, observation records, coding memos, and documentation of the data analysis process, allowing for transparency and logical traceability throughout the study. Finally, confirmability was maintained through repeated comparison of coded data with original transcripts, ongoing reflexive engagement with the dataset, and expert review of analytical interpretations. Member checking was also conducted, wherein selected participants reviewed their transcripts to verify accuracy and provide clarification when needed. For example, one participant added during verification: “*It’s not only the lack of equipment—what matters more is that when we are busy, we cannot locate it quickly*.” These strategies helped ensure that the findings, interpretations, and conclusions were grounded in the participants’ accounts rather than researcher bias.

### 2.6 Ethical considerations

This study was conducted in accordance with the Declaration of Helsinki, and approved by the Ethics Committee of the Affiliated Hospital of Zunyi Medical University (Approval number: KLL-2024-588, 20/2/2025). Prior to data collection, research participants were informed of the study’s purpose and intended use, and explicit, documented informed consent was obtained from all participants. The research process was entirely voluntary, anonymous, and confidential. All recordings and transcribed texts utilized anonymous identifiers (e.g., N1, N2) to replace any personal information. In the final paper and any research outputs, we will remove all details that could potentially reveal participant identities. All audio files and original transcripts containing identifiers are stored on password-protected encrypted hard drives. All interviews were conducted in completely isolated, soundproof private rooms to ensure conversations could not be overheard by third parties. Participants were explicitly informed of their right to refuse any question and to pause or withdraw from the interview at any time without condition.

## 3 Results

The sample size was determined based on the principle of information saturation. Ultimately, 14 participants were recruited, including 10 specialized nurses with specialized nursing qualifications. The participants’ ages ranged from 32 to 55 years old (mean age: 39.64 ± 6.18 years), and their average years of experience in anesthesia-related nursing fields was 5.79 ± 3.40 years. The detailed characteristics of the participants are shown in Table 1.

**TABLE 1:** Characteristics of interviewed participants (*n* = 14).

Number	Gender	Age (years)	Years of experience	Years of experience (anesthetic nursing)	Anesthesia nurse specialist	Education level
N1	Female	55	35	3	No	Bachelor’s degree
N2	Female	39	18	8	Yes	Bachelor’s degree
N3	Female	35	13	5	Yes	Bachelor’s degree
N4	Female	38	15	4	Yes	Postgraduate
N5	Female	34	12	1	No	Bachelor’s degree
N6	Male	37	15	5	No	Bachelor’s degree
N7	Female	44	23	5	Yes	Bachelor’s degree
N8	Female	35	12	12	Yes	Bachelor’s degree
N9	Female	45	24	10	Yes	Bachelor’s degree
N10	Female	42	21	4	Yes	Bachelor’s degree
N11	Female	35	15	9	Yes	Bachelor’s degree
N12	Female	46	25	10	Yes	Bachelor’s degree
N13	Female	38	15	3	Yes	Bachelor’s degree
N14	Female	32	11	2	No	Bachelor’s degree

N(nurse).

Based on the PRECEDE-PROCEED model, three overarching categories and nine subthemes were identified from the interviews, reflecting the actual experiences of PACU nurses’ hypothermia prevention behaviors. These include: predisposing factors, enabling factors, and reinforcing factors. A complete encoding tree has been constructed, which is provided in Supplementary Table 1 with illustrative quotes.

### 3.1 Theme 1: predisposing factors

Predisposing factors are essential cognitive or psychological elements that motivate behavior, such as knowledge, attitudes, beliefs, preferences, skills, and self-efficacy (14). When anesthetic nursing staff lack sufficient knowledge about the prevention of intraoperative hypothermia, have misconceptions about the subject, or have low self-efficacy, their willingness and behavioral compliance in actively implementing temperature protection measures during the anesthetic recovery period are significantly weakened.

#### 3.1.1 Knowledge gaps on hypothermia

Most anesthetist nurses place a high priority on hypothermia prevention management and proactively provide patients with basic and comprehensive hypothermia management. However, nurses also acknowledge that hypothermia prevention requires more specialized knowledge and skills. The lack of professional knowledge and skills is one of the factors affecting their ability to implement hypothermia prevention measures. For example, N1 and N11 stated: “I know hypothermia affects recovery, but I don’t really understand the specific harms or the guidelines.” N2 noted “We lack knowledge on how to effectively prevent hypothermia. If we really want to prevent hypothermia, we need to be more specific.” N7 shared “I’m not really sure about the guidelines like that.” N12 added: “I’ve heard of guidelines on hypothermia, but I haven’t studied them in detail.”

#### 3.1.2 Complexity of evidence-to-practice translation

Guidelines and consensus statements in related fields provide evidence-based guidance for the prevention and management of hypothermia in patients undergoing anesthesia and recovery. Some participants found clinical application of evidence-based guidelines complicated and time-consuming.

N4 noted: “Hypothermia prevention requires continuous management before, during, and after surgery. The application of evidence requires many steps and multidisciplinary collaboration to assess quality and obtain expert consultation before use, which is a relatively complex process.” N8 stated: “I learned about evidence appraisal in a conference, but quality evaluation is too specialized for me to achieve. But it’s hard to apply in practice.”

#### 3.1.3 Conflicts between mindset and habitual practices

Some nurses noted that existing workflows did not align well with recommended hypothermia management practices because of hypothermia prevention management conflicts with clinical work habits. N3 noted: “Our protocol is well written, but not every part is followed in practice.” N11 shared: “I learned procedures and guidelines related to hypothermia, but in actual work, I don’t have time to follow the procedures for assessment, and most of the time I rely on my experiences.”

### 3.2 Theme 2: enabling factors

Enabling factors are essential elements that facilitate behavioral change and influence the occurrence of target behaviors (14), including resources, training, or equipment availability. Most PACU nurses believe that even with proper knowledge, it remains challenging to ensure the implementation of hypothermia prevention measures when relevant promoting factors are lacking.

#### 3.2.1 Insufficient human and equipment resources

Some respondents indicated that nurses are the primary implementers of hypothermia prevention measures. Adequate human resources are essential for the effective implementation of hypothermia prevention measures during the anesthesia recovery period. N10 said: “I believe that having sufficient human resources is a crucial factor in promoting hypothermia prevention efforts.” Some respondents believed that hypothermia management devices were the main factors promoting improved hypothermia prevention practices, such as inflatable warming devices and intravenous fluid warming devices. N12 shared: “Sometimes we need to use heating equipment at the same time, but we don’t have enough equipment or cannot be immediately identified, so there is not enough to go around, which affects the implementation of hypothermia prevention measures.”

#### 3.2.2 Lack of targeted and continuous training

Most nurses indicated a lack of continuous, targeted training on hypothermia, resulting in limited knowledge and operational skills that do not meet clinical practice needs. N2 shared: “I learned some things during a specialist nurse course, but after returning to work, I occasionally discussed the topic during rounds at the hospital.” N5 remarked: “I have not received formal training on hypothermia, but I occasionally see some related information on relevant platforms.” Without professional training and guidance, it is difficult to achieve systematic, comprehensive, and in-depth learning outcomes.” N14 said: “We only touch on hypothermia occasionally in case reviews-I’d like more professional, guideline-based training.”

#### 3.2.3 Lack of standardized guidelines for hypothermia prevention management processes

Some respondents pointed out that standardized procedures can strengthen standardized temperature management for patients in the recovery period, reduce the incidence of hypothermia-related complications, and optimize postoperative outcomes for patients. N5 shared: “I believe that uniform hypothermia prevention standards and procedures can help me implement hypothermia prevention.” N12 stated: “When I encounter older adult people or children, I pay special attention to preventing hypothermia. There is no fixed method or procedure for doing this; I just follow my own clinical experience.” N13 said: “There’s no tool to identify high-risk patients. A standardized assessment would help.” N9 remarked: “Sometimes I touch the patient’s hand, and if it feels cold, I will keep them warm.” N7 explained: “If there were standardized guidelines for hypothermia prevention and management, I would change my previous practices and implement temperature management.”

#### 3.2.4 Limitations in hospital information systems and lack of intelligent monitoring functions

Some interviewees believed that the lack of intelligence in hospital information systems hindered the continuous assessment of hypothermia prevention.

N12 said: “BMI is an important risk assessment indicator, but the system cannot automatically obtain and calculate the patient’s BMI value. If height and body weight data can be automatically captured and calculated, it will significantly improve the efficiency and accuracy of the assessment.” N10 noted: “In the context of intelligent healthcare, existing systems lack intelligent temperature monitoring capabilities and are unable to automatically capture patient clinical data and dynamically identify risk factors.”

### 3.3 Theme 3: reinforcing factors

Reinforcing factors are factors that promote the continuation or reduction of target behaviors (11), including external support and supervision. When PACU nurses lack a collaborative team atmosphere or have not established a supportive work environment and supervision mechanisms for hypothermia prevention.

#### 3.3.1 Healthcare collaboration and peer support promote hypothermia prevention practices

Some interviewees believed that medical and nursing cooperation could promote the implementation of hypothermia prevention management. They believed that communicating and learning from experienced colleagues could help them standardize the implementation of hypothermia prevention. N14 shared: “On-site guidance from senior medical staff can effectively standardize my temperature management procedures, especially when it comes to choosing warming strategies for patients undergoing surgery in special positions.” N13 said: “Hypothermia prevention should be established by an MDT (Multidisciplinary Team) temperature management team’, which requires preoperative assessment, intraoperative and postoperative warming, and involves complex and diverse influencing factors. Doctors, nurses, and anesthesiologists should jointly participate in patient assessment, formulate plans, and then jointly implement them.”

#### 3.3.2 Lack of supervisory network for hypothermia prevention

In 2022, the National Anesthesia Quality Control Center included the incidence of hypothermia during general anesthesia surgery and the incidence of hypothermia upon admission to the recovery room as core indicators for anesthesia medical quality control (7). However, some interviewees pointed out that hypothermia prevention practices face issues such as “no quantitative standards, difficult supervision, and quality fluctuations.” N3 remarked: “Continuous monitoring is key, but there are no clear metrics or supervisory mechanisms.”

N10 said: “Currently, low-temperature prevention measures are limited to checking items on nursing records. There is no automatic collection of dynamic body temperature monitoring data, nor is there anyone specifically responsible for verifying the accuracy of the measures implemented.” N11: “Key parameters such as whether the temperature settings in the recovery room are up to standard and the integrity of patient surface coverage lack objective recording methods in the existing system. I believe that a professional quality control nurse can improve overall quality.”

## 4 Discussion

This study employed a theory-based qualitative approach to summarize the actual experiences of nurses in the PACU regarding the implementation of hypothermia prevention practices. Using the PRECEDE-PROCEED model as the analytical framework, we identified multiple interrelated factors across three domains: predisposing, enabling, and reinforcing factors. These findings echo previous international research on behavioral change among clinical nurses (10, 20), and provide a culturally relevant understanding of hypothermia management in the PACU context.

Predisposing factors, as the theoretical basis of behavior change, serve as the prerequisite conditions and intrinsic motivators that facilitate behavioral initiation (14). Findings from this study indicate that insufficient knowledge regarding hypothermia prevention directly contributes to nurses’ limited understanding of the importance of such behaviors. As a result, nurses may fail to recognize the potential risks and adverse consequences of hypothermia for patients. This knowledge gap may trigger a chain reaction manifested as unclear behavioral goals, diminished self-efficacy, and reduced risk awareness, ultimately hindering the implementation of prevention behaviors. This mechanism aligns with the core principles of evidence-based nursing, wherein professional knowledge forms the foundation of standardized clinical practice. The inhibitory role of knowledge deficits on behavior has been repeatedly confirmed in multiple national and international studies focusing on nursing behaviors across various specialties (21, 22). Findings from this study revealed that the complexity of translating evidence into practice is a key factor hindering the standardization of hypothermia prevention behaviors, which is consistent with similar reports in the literature (23). This challenge is mainly attributed to nurses’ limited knowledge and suboptimal attitudes toward evidence-based nursing practice (24). The gap identified between theoretical knowledge and clinical application reflects a “knowledge–practice disconnect,” suggesting not only individual cognitive limitations but also systemic deficiencies in the cultivation of evidence-based thinking within nursing education. The challenge of evidence translation appears to be more pronounced in the Chinese clinical nursing context, likely due to the later introduction and slower integration of evidence-based practice in nursing curricula. Therefore, strengthening professional development remains crucial. Future efforts should focus on enhancing anesthesia nurses’ education, training, and continuous professional development to improve both the depth and breadth of specialty knowledge. Additionally, with the increasing demand for high-quality patient outcomes, nurses should also broaden their interdisciplinary knowledge base to better address complex clinical needs and deliver patient-centered care.

Enabling factors refer to external conditions that support or hinder the execution of target behaviors (14). In this study, most participants identified staffing adequacy, standardized training, and workflow protocols as primary enabling factors, which is consistent with the findings of Drayton et al. (25). Adequate human resources can reduce workload burden and serve as a fundamental condition for ensuring care quality, enhancing service delivery, improving resource allocation efficiency, and increasing patient satisfaction (26, 27). Several participants further indicated that sufficient staffing allowed them to implement hypothermia prevention strategies in a more standardized and consistent manner. With the rapid advancement of medical technology and increasing diversity in patient needs, national standards have placed greater emphasis on the training and deployment of specialized nurses. Anesthesia nursing practice encompasses management, clinical care, teaching, and research, making it essential for strengthening competency development among anesthesia nurses (28). However, the high workload commonly reported among participants indicates a persistent strain in clinical practice. Evidence suggests that excessive workload is strongly associated with professional burnout among anesthesia nurses (29). Our study also emphasizes the importance of adequate technical support, such as warming devices and intelligent decision-support systems. According to the national “Operating Room Medical Equipment Configuration Standards” (30), the ratio of warming devices to operating rooms in tertiary hospitals should be at least 1:3. Additionally, with the advancement of electronic medical records and artificial intelligence, clinical decision support systems have gradually been applied in the field of anesthesia. These systems can quickly convert complex and disorganized patient information into concise and organized data for analysis, thereby providing support for clinical decision-making (31, 32). Therefore, it is recommended that hospital administrators optimize clinical staffing, provide specialized anesthesia nursing training based on needs, establish a hypothermia management team, integrate existing best evidence, develop standardized procedures based on actual conditions, and utilize artificial intelligence technology to construct a hypothermia prevention decision support system to facilitate the implementation of hypothermia prevention measures by anesthesia nurses.

Reinforcing factors refer to mechanisms that sustain or diminish target behaviors, such as feedback, peer support, and quality monitoring systems (14). This study identifies social support and systematic supervision as two core reinforcement mechanisms, similar to the research findings (33). In this study, supportive feedback from colleagues and supervisors served as a form of positive social reinforcement, which strengthened nurses’ behavioral intentions and enhanced their sense of team belonging. Findings indicate that multidisciplinary collaboration models led by specialty nurses not only provide such social support but also systematically improve nursing quality and patient outcomes through clear role division and a culture of collaboration, and enhance care satisfaction (34, 35). This study also found that the lack of a hypothermia prevention management supervision network is a factor contributing to the weakening of hypothermia prevention behaviors among PACU nurses. Due to limitations in research resources, certain functions of the intelligent monitoring system have not yet been implemented. Research indicates that establishing independent quality management teams composed of specialized nurses, developing a quality indicator system, and providing training can reduce the incidence of complications in patients during the anesthesia recovery period, improve the utilization rate of the anesthesia recovery period, and ensure the safety of patients during the anesthesia recovery period (36). Standardized evaluation criteria can reduce variability among institutions and support ongoing quality improvement (37). Therefore, future research suggests that nurse managers could incorporate performance metrics into quality evaluation indicators to facilitate the implementation of hypothermia-prevention measures (38). Establishing quality supervision teams within multidisciplinary frameworks, regularly auditing performance data, linking evaluation outcomes to performance-based incentives, and leveraging digital technologies to develop risk warning modules are recommended strategies. Artificial intelligence–driven clinical decision support systems (AI-CDSS) are considered key tools for optimizing diagnostic and treatment processes, as well as enhancing the efficiency and quality of healthcare services (39). Collectively, these strategies contribute to the gradual realization of an intelligent, networked, and closed-loop quality management system.

## 5 Limitations

This study also has limitations. First, all participants were drawn from the same Grade A tertiary hospital in Guizhou Province, China. While this facilitates a deeper understanding of experiences within this specific context, it may limit the transferability of the findings. Institutions with different healthcare contexts, resource allocations, or cultural environments should consider their own circumstances when applying these findings. Second, to enhance rigor, we employed member validation and dual independent coding and analysis to ensure neutrality. However, our professional background as clinical anesthesia nurses may potentially influence data interpretation. We addressed this through ongoing reflexive practices, and team discussions to examine and mitigate such biases. Furthermore, the translation of interview data from Chinese to English may involve subtle semantic loss. While we strive for accuracy, this remains an inherent challenge in cross-cultural research communication.

## 6 Conclusion

This study used a descriptive research method to summarize the actual experiences from anesthesia nurses’ implementation of hypothermia prevention measures in Guizhou Province, China. Through in-depth data analysis and refinement, the study identified key predisposing, enabling, and reinforcing factors affecting their behavior. Based on the findings, to improve hypothermia prevention in the PACU, nursing administrators should enhance anesthesia nurses’ professional knowledge through targeted training, optimize staffing and provide need-based, multidimensional education programs, ensure sufficient warming equipment and establish standardized management protocols supported by intelligent systems, and strengthen interprofessional collaboration and implement digital quality monitoring networks. These efforts will contribute to sustained behavioral improvements among PACU nurses, reduce postoperative hypothermia incidence, improve patient outcomes, and promote continuous quality improvement.

## Data Availability

The original contributions presented in this study are included in this article/Supplementary material, further inquiries can be directed to the corresponding author.

## References

[1] Giuliano K Hendricks J. Inadvertent perioperative hypothermia: current nursing knowledge. *AORN J.* (2017) 105:453–63. 10.1016/j.aorn.2017.03.003 28454611

[2] Al Farhan M Iqbal J. Reassessing postoperative hypothermia prediction: Addressing key limitations and improving clinical applicability. *J Clin Nurs.* (2025): 10.1111/jocn.70001 Online ahead of print. 40490905

[3] Bateman RM Sharpe MD Jagger JE Ellis CG Solé-Violán J López-Rodríguez M 36th International Symposium on Intensive Care and Emergency Medicine: Brussels, Belgium. 15–18 March 2016. *Crit Care*. (2016) 20(Suppl 2):94. 10.1186/s13054-016-1208-6 27885969 PMC5493079

[4] Xu R Hu X Sun Z Zhu X Tang Y. Incidence of postoperative hypothermia and shivering and risk factors in patients undergoing malignant tumor surgery: A retrospective study. *BMC Anesthesiol.* (2023) 23:31. 10.1186/s12871-023-01991-8 36690942 PMC9869522

[5] Vural F Çelik B Deveci Z Yasak K. Investigation of inadvertent hypothermia incidence and risk factors. *Turk J Surg.* (2018) 34:300–5. 10.5152/turkjsurg.2018.3992 30664429 PMC6340665

[6] Blondin N. Diagnosis and management of periodic hypothermia. *Neurol Clin Pract.* (2014) 4:26–33. 10.1212/01.CPJ.0000437350.47610.3a 29473588 PMC5765588

[7] Gu X Yi J Pei L. Expert consensus on the prevention and treatment of hypothermia in perioperative patients (2023 Edition). *J Clin Anesthesiol.* (2023) 39:764–71. 10.12290/xhyxzz.2023-0266

[8] Cenacchi C Giusti M Peghetti A Quirini S Tinelli F Giorgi S Optimization of nursing staff standards in the perioperative settings of the IRCCS university hospital of bologna. An improvement project. *Front Public Health.* (2025) 13:1601290. 10.3389/fpubh.2025.1601290 40642247 PMC12241087

[9] Sirevåg I Tjoflåt I Hansen B. A delphi study identifying operating room nurses’ non-technical skills. *J Adv Nurs.* (2021) 77:4935–49. 10.1111/jan.15064 34626011

[10] Feng L Han X Zhang C Zhang Q. A qualitative study on the factors influencing nurses’ hypothermia prevention behaviors during cesarean section. *Chinese J Nurs.* (2025) 60:1190–5. 10.3870/j.issn.1001-4152.2020.23.031

[11] Munday J Delaforce A Forbes G Keogh S. Barriers and enablers to the implementation of perioperative hypothermia prevention practices from the perspectives of the multidisciplinary team: a qualitative study using the theoretical domains framework. *J Multidiscip Healthc.* (2019) 12:395–417. 10.2147/JMDH.S209687 31239694 PMC6551587

[12] Ironside P. Using narrative pedagogy: learning and practising interpretive thinking. *J Adv Nurs.* (2006) 55:478–86. 10.1111/j.1365-2648.2006.03938.x 16866843

[13] Doyle L McCabe C Keogh B Brady A McCann M. An overview of the qualitative descriptive design within nursing research. *J Res Nurs.* (2020) 25:443–55. 10.1177/1744987119880234 34394658 PMC7932381

[14] Kim J Jang J Kim B Lee K. Effect of the PRECEDE-PROCEED model on health programs: a systematic review and meta-analysis. *Syst Rev.* (2022) 11:213. 10.1186/s13643-022-02092-2 36210473 PMC9549687

[15] Crosby R Noar S. What is a planning model? An introduction to PRECEDE-PROCEED. *J Public Health Dent.* (2011) 71:S7–15. 10.1111/j.1752-7325.2011.00235.x 21656942

[16] Assarroudi A Heshmati Nabavi F Armat M Ebadi A Vaismoradi M. Directed qualitative content analysis: the description and elaboration of its underpinning methods and data analysis process. *J Res Nurs.* (2018) 23:42–55. 10.1177/1744987117741667 34394406 PMC7932246

[17] Tong A Sainsbury P Craig J. Consolidated criteria for reporting qualitative research (COREQ): a 32-item checklist for interviews and focus groups. *Int J Qual Health Care.* (2007) 19:349–57. 10.1093/intqhc/mzm042 17872937

[18] Braun V Clarke V. Using thematic analysis in psychology. *Qual Res Psychol.* (2006) 3:77–101. 10.1191/1478088706qp063oa 32100154

[19] Lincoln Y Guba E. But is it rigorous? Trustworthiness and authenticity in naturalistic evaluation. *New Dir Program Eval.* (1986) 1986:73–84. 10.1002/ev.1427

[20] Huang L Liu M Leung A Zhang J Deng R Dai H Exploring the influencing factors of experienced nurses’ retention behaviour: a qualitative study based on the COM-B model. *J Adv Nurs.* (2025) 81:4122–35. 10.1111/jan.16611 39526439

[21] Lang H Zhang X Yan N Du J Jiang X. Knowledge, attitude, and belief of healthcare professionals toward obesity stigmatization. *J Multidiscip Healthc.* (2025) 18:1935–46. 10.2147/JMDH.S499828 40224909 PMC11988193

[22] Høghaug G Skår R Tran T Schou-Bredal I. Nurses’ experiences with newly acquired knowledge about medication management: a qualitative study. *J Nurs Manag.* (2019) 27:1731–7. 10.1111/jonm.12864 31495022

[23] Zhang X Qiu J Tang L Xing W. Factors contributing to lipid management challenges in breast cancer patients: an implementation research framework approach. *Chinese J Nurs Manag.* (2025) 25:742–7. 10.3969/j.issn.1672-1756.2025.05.020

[24] Fu Y Wang C Hu Y. Content analysis of barriers and facilitators in the clinical translation of evidence. *J Nurs Sci.* (2022) 29:64–8. 10.16460/j.issn1008-9969.2022.05.064

[25] Drayton N Waddups S Walker T. Exploring distraction and the impact of a child life specialist: perceptions from nurses in a pediatric setting. *J Spec Pediatr Nurs.* (2019) 24:e12242. 10.1111/jspn.12242 30901151

[26] Lv Y Ge B Yu Y. Current status of nursing human resource allocation in Guizhou province. *Nurs Res.* (2025) 39:2011–8. 10.12102/j.issn.1009-6493.2025.12.007

[27] Du M Wang H Liu Y Zeng T Wang Y Li M A review of the scope of evaluation indicators for nursing human resource allocation. *Chinese J Nurs.* (2023) 58:366–73. 10.3761/j.issn.0254-1769.2023.03.015

[28] Fan Z Ding H Li Y Huang J Xiao L Zhang G. Development and preliminary application of evaluation criteria for establishing clinical training bases for anesthesia nurse practitioners. *J Nurs Sci.* (2024) 39:51–5. 10.3870/j.issn.1001-4152.2024.16.051

[29] Li Y Xiang D Jiang Y Gu S. Understanding burnout among operating room nurses: a qualitative study. *Front Public Health.* (2025) 13:1604631. 10.3389/fpubh.2025.1604631 40491999 PMC12146190

[30] National Health Commission of the People’s Republic of China. *Standards for the Configuration of Medical Equipment in Operating Rooms.* (2025). Available online at: https://www.nhc.gov.cn/wjw/c100308/202404/9200508723274d0795f0d78dd2b6babc.shtml (accessed August 4, 2025)

[31] Freundlich R Barnet C Mathis M Shanks A Tremper K Kheterpal S. A randomized trial of automated electronic alerts demonstrating improved reimbursable anesthesia time documentation. *J Clin Anesth.* (2013) 25:110–4. 10.1016/j.jclinane.2012.06.020 23333782

[32] Bihorac A Ozrazgat-Baslanti T Ebadi A Motaei A Madkour M Pardalos P MySurgeryRisk: Development and validation of a machine-learning risk algorithm for major complications and death after surgery. *Ann Surg.* (2019) 269:652–62. 10.1097/SLA.0000000000002706 29489489 PMC6110979

[33] McPherson S Mitchell A Sletten I Kvande M Steindal S. Haematological nurses’ experiences about palliative care trajectories of patients with life-threatening haematological malignancies: a qualitative study. *Nurs Open.* (2023) 10:3094–103. 10.1002/nop2.1558 36539384 PMC10077405

[34] Wei J Fang X Qiao J Liu H Cui H Wei Y Construction on teaching quality evaluation indicator system of multi-disciplinary team (MDT) clinical nursing practice in China: a delphi study. *Nurse Educ Pract.* (2022) 64:103452. 10.1016/j.nepr.2022.103452 36152471

[35] Ryan T Harrison M Gardiner C Jones A. Challenges in building interpersonal care in organized hospital stroke units: the perspectives of stroke survivors, family caregivers and the multidisciplinary team. *J Adv Nurs.* (2017) 73:2351–60. 10.1111/jan.13313 28378452

[36] Cheng Z Wang Y Li J Wang X Guo Q. Strengthening the management of the anesthesia recovery room to improve perioperative patient safety. *J Clin Anesthesiol.* (2021) 37:5–8. 10.12089/jca.2021.01.001

[37] Liu M Guo R Li J Wang C Yu L Liu M. Process indicators outshine outcome measures: assessing hospital quality of care in breast cancer treatment in China. *Sci Rep.* (2024) 14:19137. 10.1038/s41598-024-70474-8 39160221 PMC11333708

[38] He W Du L Zhang W. Development of best performance evaluation indicators based on value-based healthcare for general hospital nursing: a delphi study. *J Clin Nurs.* (2025) 34:816–25. 10.1111/jocn.17405 39152561

[39] Yu Z Hu N Zhao Q Hu X Jia C Zhang C The willingness of doctors to adopt artificial intelligence-driven clinical decision support systems at different hospitals in China: Fuzzy set qualitative comparative analysis of survey data. *J Med Internet Res.* (2025) 27:e62768. 10.2196/62768 39773696 PMC11751641

