# Supplementary material for: The actual experiences of nurses’ in implementing hypothermia prevention practices during post-anesthesia care unit: a qualitative study based on the PRECEDE-PROCEED model
Source: Front Med (Lausanne). 2025 Dec 10;16:1687814. doi: 10.3389/fmed.2025.1687814 (PMC12727933; doi:10.3389/fmed.2025.1687814)
Supplement: Supplementary file 1 [file Table_1.pdf]

Supplementary Table 1. Themes, Subthemes, and Illustrative Quotes

Main themes	Sub-themes	Illustrative quotes
Predisposing Factors	Knowledge gaps on hypothermia	“I know hypothermia affects recovery, but I don’t really understand the specific harms or the guidelines.”(N1 and N11)<br>“We lack knowledge on how to effectively prevent hypothermia. If we really want to prevent hypothermia, we need to be more specific.”（N2）<br>“I'm not really sure about the guidelines like that."（N7）<br>“I’ve heard of guidelines on hypothermia, but I haven’t studied them in detail.”（N12）
	Complexity of evidence-to-practice translation	“ Hypothermia prevention requires continuous management before, during, and after surgery. The application of evidence requires many steps and multidisciplinary collaboration to assess quality and obtain expert consultation before use, which is a relatively complex process.”（N4）<br>“I learned about evidence appraisal in a conference, but quality evaluation is too specialized for me to achieve.but it’s hard to apply in practice.”（N8）
	Conflicts between mindset and habitual practices	“Our protocol is well written, but not every part is followed in practice.”（N3）<br>“ I learned procedures and guidelines related to hypothermia, but in actual work, I don't have time to follow the procedures for assessment, and most of the time I rely on my expericence.”（N11）
Enabling Factors	Insufficient human and equipment resources	“I believe that having sufficient human resources is a crucial factor in promoting hypothermia prevention efforts.”（N10）<br>“Sometimes we need to use heating equipment at the same time, but we don't have enough equipment, so there is not enough to go around, which affects the implementation of hypothermia prevention measures.”（N12）
	Lack of targeted and continuous training	“I learned some things during a specialist nurse course, but after returning to work, I occasionally discussed the topic during rounds at the hospital.”（N2 ）<br>“ I have not received formal training on hypothermia, but I occasionally see some related information on relevant platforms. Without professional training and guidance, it is difficult to achieve systematic, comprehensive, and in-depth learning outcomes.”（N5 ）<br>“ We only touch on hypothermia occasionally in case reviews-I’d like more professional, guideline-based training.”（N14 ）

	Lack of standardized guidelines for hypothermia prevention management processes	“I believe that uniform hypothermia prevention standards and procedures can help me implement hypothermia prevention.”（N5）<br>“When I encounter elderly people or children, I pay special attention to preventing hypothermia. There is no fixed method or procedure for doing this; I just follow my own clinical experience.”（N12）<br>“There’s no tool to identify high-risk patients. A standardized assessment would help.”（N13）<br>Sometimes I touch the patient's hand, and if it feels cold, I will keep them warm.”（N9）<br>“If there were standardized guidelines for hypothermia prevention and management, I would change my previous practices and implement temperature management.””（N7）
	Limitations in hospital information systems and lack of intelligent monitoring functions	“BMI is an important risk assessment indicator, but the system cannot automatically obtain and calculate the patient's BMI value. If height and body weight data can be automatically captured and calculated, it will significantly improve the efficiency and accuracy of the assessment.”（N12）<br>“In the context of intelligent healthcare, existing systems lack intelligent temperature monitoring capabilities and are unable to automatically capture patient clinical data and dynamically identify risk factors.”（N10）
Reinforcing Factors	Healthcare collaboration and peer support promote hypothermia prevention practices	“On-site guidance from senior medical staff can effectively standardize my temperature management procedures, especially when it comes to choosing warming strategies for patients undergoing surgery in special positions.”（N14）<br>“Hypothermia prevention should be established by an ‘MDT temperature management team’, which requires preoperative assessment, intraoperative and postoperative warming, and involves complex and diverse influencing factors. Doctors, nurses, and anesthesiologists should jointly participate in patient assessment, formulate plans, and then jointly implement them.”（N13）
	Lack of supervisory network for hypothermia prevention	“Continuous monitoring is key, but there are no clear metrics or supervisory mechanisms.”（N3）<br>“Currently, low-temperature prevention measures are limited to checking items on nursing records. There is no automatic collection of dynamic body temperature monitoring data, nor is there anyone specifically responsible for verifying the accuracy of the measures implemented.”（N10）<br>“Key parameters such as whether the temperature settings in the recovery room are up to standard and the integrity of patient surface coverage lack objective recording methods in the existing system. I believe that a professional quality control nurse can improve overall quality.”（N11）
